# PSMA-PET response under [^177^Lu]Lu-PSMA therapy: Comparison of RECIST, PERCIST, PPP, adapted PCWG4 and RECIP criteria

**DOI:** 10.1007/s00259-026-07901-7

**Published:** 2026-04-24

**Authors:** Tugce Telli, Madeleine J. Karpinski, Robert Seifert, Andrei Gafita, Caner Civan, Ayelet Walter, Viktor Grünwald, Boris Hadaschik, Claudia Kesch, Lale Umutlu, Ken Herrmann, Wolfgang P. Fendler

**Affiliations:** 1 Department of Nuclear Medicine, University Hospital Essen, Hufelandstraße 55, 45147 Essen, Germany; 2 German Cancer Consortium (DKTK), Partner Site, Essen–Düsseldorf, Partnership Between DKFZ and University Hospital Essen, Essen, Germany; 3 West German Cancer (WTZ), Essen, Germany; 4 Department of Nuclear Medicine, Inselspital Bern, Bern, Switzerland; 5 Division of Nuclear Medicine and Molecular Imaging, Russell H. Morgan Department of Radiology and Radiological Science, Johns Hopkins University School of Medicine, Baltimore, MD USA; 6 Carolus Institute for Urologic Oncology, University Hospital Essen, Essen, Germany; 7 Department of Urology, University Hospital Essen, Essen, Germany; 8 Department of Diagnostic and Interventional Radiology and Neuroradiology, University Hospital Essen, Essen, Germany

**Keywords:** PSMA, [^177^Lu]Lu-Prostate-specific membrane antigen therapy, Therapy response, Prostate cancer

## Abstract

**Purpose:**

We aimed to compare various imaging-based response criteria in men with metastatic castration-resistant prostate cancer (mCRPC) treated with [^177^Lu]Lu-Prostate-specific membrane antigen radioligand therapy (LuPSMA).

**Methods:**

This retrospective study included 84 men who received a median of 4 [^177^Lu]PSMA cycles (IQR 2–5) and median of 24.3 GBq (IQR 14.9–32.9 GBq) at the Department of Nuclear Medicine at University Hospital Essen between March 2019 and May 2022. Response assessments were conducted comparing baseline PET/CT and PET/CT at 6–8 weeks after second cycle of LuPSMA using multiple criteria: Response Evaluation Criteria in Solid Tumors (RECIST) 1.1, the adapted Prostate Cancer Working Group Criteria 4 (aPCWG4) without follow-up confirmation, Positron Emission Tomography Response Criteria in Solid Tumors (PERCIST), the PSMA PET Progression (PPP), and Response Evaluation Criteria in PSMA-Imaging 1.0 (RECIP) with visual assessment or different quantitative volumetry methods (qPSMA, SUV ≥ 4). Responses were categorized as progressive disease (PD) or non-PD. The primary endpoint was the prognostic significance of these response criteria for overall survival, evaluated via Cox regression analysis. Harrell`s C-index was used for concordance of different imaging-based criteria and survival.

**Results:**

A total of 34 (40.5%) patients had non-measurable disease according to RECIST. Twenty (40%), 41 (48.8%), 44 (52.4%), 47 (56.0%), 35 (41.7%), 37 (44.0%), and 31 (36.9%) of patients had PD according to RECIST 1.1, aPCWG4, PERCIST, PPP, RECIP qPSMA, RECIP SUV ≥ 4, and visual RECIP respectively. PD patients had a significantly higher risk of death compared to non-PD patients according to all response criteria as follows: RECIST 1.1 (HR = 5.0; 95%CI, 2.5–10.0; p < 0.001), PERCIST (HR = 2.9; 95%CI, 1.8–4.8; p < 0.001), aPCWG4 (HR = 3.6; 95%CI, 2.1–6.0; p < 0.001), PPP (HR = 3.9; 95%CI, 2.3–6.6; p < 0.001), RECIP qPSMA (HR = 2.7; 95%CI, 1.6–4.4; p < 0.001), RECIP SUV ≥ 4 (HR = 2.2; 95%CI, 1.6–3.6; p < 0.001) and visual RECIP (HR = 3.5; 95%CI, 2.1–6.0; p < 0.001). Highest C-indices were observed for PPP: 0.68 (95%CI, 0.58–0.77), and aPCWG4: 0.67 (95%CI, 0.57–0.77) in comparison to PERCIST: 0.65 (95%CI, 0.54–0.75), visual RECIP: 0.63 (95%CI, 0.52–0.73), RECIP qPSMA: 0.62 (95%CI, 0.51–0.74) and, RECIP SUV ≥ 4: 0.62 (95%CI, 0.50–0.73).

**Conclusion:**

Disease progression during [^177^Lu]PSMA in interim PET imaging, as indicated by any of the evaluated criteria, was associated with elevated risk of death. PSMA PET Progression (PPP) and aPCWG4 demonstrated the highest concordance with overall survival whereas RECIP had lowest PD rate. These findings support the prognostic value and consistency of PSMA-PET response assessment during [^177^Lu]PSMA and highlight the need for standardized, reproducible longitudinal response criteria and support their validation in large, prospective multicenter studies.

**Supplementary Information:**

The online version contains supplementary material available at 10.1007/s00259-026-07901-7.

## Introduction

Metastatic castration-resistant prostate cancer (mCRPC) represents the advanced stage of prostate cancer, characterized by progression despite castrate levels of testosterone. The emergence of novel systemic therapies, such as androgen receptor signaling inhibitors, chemotherapy, and targeted radioligand therapies like [^177^Lu]Lu-Prostate-specific membrane antigen radioligand therapy (LuPSMA) has expanded treatment options and improved survival outcomes in mCRPC patients [1]. In this evolving therapeutic landscape, accurate restaging is critical for detecting progression and guiding patient selection for specific therapies. Conventional imaging modalities—such as bone scintigraphy, CT, and MRI—are standard of care according to Prostate Cancer Working Group 3 (PCWG3) guidelines and have been used in all registrational studies in the last decade [2]. However, these approaches have limited sensitivity and specificity, particularly in the assessment of bone and small nodal metastases [3].

As precision medicine approaches start to change prostate cancer management, PSMA positron emission tomography/computed tomography (PET/CT) may help to personalize treatment, monitor therapeutic efficacy, and improve clinical outcomes in patients with mCRPC offering a promising solution to overcome the limitations of conventional imaging. PSMA PET/CT enables comprehensive assessment of tumor burden, early detection of disease progression, and improved patient stratification, supporting its integration into clinical trials and routine practice for monitoring LuPSMA therapy [1, 4–7]. Several response criteria for PSMA PET have been proposed. The PSMA PET progression (PPP) criteria defines progression as the appearance of at least 2 new lesions – among other parameters, which mirrors the PCWG3 criteria for progression on bone scan [8]. Response on interim PSMA PET/CT has emerged as a robust biomarker for monitoring treatment efficacy and predicting overall survival in patients undergoing LuPSMA therapy. The RECIP 1.0 framework, based on both change in PSMA-positive total tumor volume (PSMA-VOL) and the appearance of new lesions on interim PSMA PET, enables classification for complete response (CR), partial response (PR, ≥ 30% PSMA-VOL decrease without new lesions), stable disease (SD), or progressive disease (PD, ≥ 20% PSMA-VOL increase with new lesions) [9]. However, quantitative implementation faces significant challenges: most segmentation tools are proprietary and not freely available, while comprehensive whole-body tumor burden quantification remains laborious. Moreover, although RECIP 1.0 initially defined optimal cutoffs (≥ 30% volume decrease for PR; ≥ 20% increase with occurance of new lesions for PD), these thresholds lack prospective validation in monitoring other therapy settings such as after taxan-based chemotherapy [10].

RECIP 1.0 assessments can be performed either visually by trained physicians or quantitatively through tumor segmentation software. Both approaches have shown high interreader agreement (83% vs. 92%), and concordance between visual and quantitative reads in around 95% of cases [11]. Neverthless, workflow barriers and methodological variability, particularly with regard to segmentation thresholds and tool availability, limit the widespread clinical adoption of quantitative PET assessments. Therefore, the aim of the current study was to compare various visual and volumetry-related imaging-based response criteria in men with mCRPC undergo treatment with LuPSMA.

## Methods

### Patient cohort

**Fig. 1:**
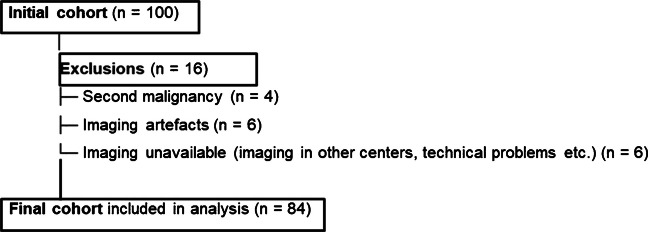
Flow diagram for the study participants

This retrospective study included 84 patients who received at least two cycles of LuPSMA cycles at the University Hospital Essen between March 2019 and May 2022 (Fig. 1)*.* As part of the clinical routine, all patients underwent an individualized assessment after baseline and interim PET scans. Further treatment decisions were made within a multidisciplinary setting. The decision to continue LuPSMA treatment or transition to alternative systemic therapies was based on clinical symptoms, patient fitness, PSA kinetics, haematological and biochemical parameters, and overall disease burden.

Eligibility criteria for this retrospective study included: 1) mCRPC, 2) availability of PSMA-PET/CT at baseline (bPET) and after two cycles of LuPSMA (interim PET/CT [iPET]), 3) availability of survival data. Clinical status and laboratory values were collected at baseline and until 12 weeks after last therapy.

Patients were followed until March 2025 to assess overall survival. The study was conducted in accordance with the Declaration of Helsinki. The local ethics committee approved this retrospective study (Permission Numbers: 23–11,501-BO and 22–10,822-BO/23–11,654) and 23–11,654-BO) waived the need for study-specific consent.

### Imaging acqusition

bPETs were performed one to four weeks before the first LuPSMA cycle whereas iPETs were performed six to eight weeks after the second LuPSMA cycle. As part of the clinical routine, PET/CT image acquisitions using either [⁶⁸Ga]Ga-PSMA-11 or [^18^F]PSMA-1007 were initiated 60 and 90–120 min after intravenous injection, respectively. In total, 56 (66.7%) bPET and 31 (36.9%) iPET scans were obtained using [⁶⁸Ga]Ga-PSMA-11, and 28 (33.3%) bPET and 53 (63.1) iPET scans using [^1^⁸F]PSMA-1007. For 49 (58.3%) of patients, the same tracer (26 [⁶⁸Ga]Ga-PSMA-11 pairs and 23 [^1^⁸F]PSMA-1007 pairs) was used at baseline and interim PET, ensuring consistency across time points. In cases where the tracer differed between bPET and iPET, no systematic differences in tracer choice were present; variations resulted from scanner availability and radiotracer supply rather than clinical factors. Images were acquired using either Siemens Biograph 128 mCT or Biograph 64 VISION 600. CT scans were performed as diagnostic scans with the application of intravenous contrast if not contraindicated. The CT scan was performed from the vertex to the mid-thigh, followed by the PET acquisition. Acquisitions and reconstructions were obtained in accordance with EANM guideline [12].

### Image analyses and therapy response assessment

All visual and semiquantitative assessments of bPET and iPET were carried out independently and blinded by a nuclear medicine physician (TT) with almost ten years of experience in PSMA PET interpretation. Consensus reading was employed by two experienced readers (TT and WF) for those patients with inconclusive results. Pathological findings on bPSMA images were assessed in terms of local disease (T), pelvic lymph node metastases (N), distant lymph node metastases (M1a), bone metastases (M1b) and visceral metastases (M1c).

Table 1 summarizes the definitions of the response criteria. For the semiquantitative therapy response assessment, semi-automated segmentation and volumetry were applied by one experienced reader (TT). Two different cutoffs were used: qPSMA as defined by the Response Evaluation Criteria in PSMA-Imaging 1.0 (RECIP), using PARS and MICIIS 1.0 Softwares (RECIP qPSMA), and an absolute cutoff of SUV ≥ 4 using LifeX v7.7.0 Software (RECIP SUV4) [13–17]. For soft tissue lesions that cannot be separated automatically from the physiological uptake of surrounding tissue, manual/adjusted contouring was used. Changes in tumor volume were evaluated as defined in RECIP 1.0. Patients were categorized as either PD or non-PD.

**Table 1:** Definitions of different response criteria

Criteria	Ref	Response	Definition
**New lesion**			PET: Visually a new focal uptake higher than background or with SUVmax > blood pool CT: Appearance of new measurable lesion on CT reflects either metastatic spread or new tumor foci
**RECIP 1.0**	[13]	**CR**	Absence of any PSMA uptake on follow-up PET
		**PR**	≥ 30% decrease in PSMA-VOL **without** any new lesion
		**SD**	< 30% decrease or < 20 increase in PSMA-VOL with or without new lesions, ≥ 30% decrease with new lesions, ≥ 20% increase in PSMA-VOL without new lesions
		**PD**	≥ 20% increase in PSMA-VOL **with** new lesions
**RECIP Visual**	[20]		Visual evaluation of changes in tumor volume as recommended by RECIP 1.0
**PPP**	[8]	**PD**	≥ 2 new PSMA positive lesions ≥ 1 new PSMA positive lesion **with** consistent clinical and/or biochemical progress or progress in follow-up imaging ≥ 1 lesion with ≥ 30% increase in size **with** consistent clinical and/or biochemical progress or progress in follow-up imaging
**aPCWG4**	[19]	**PD**	≥ 2 new PSMA positive lesions
**PERCIST**	[18]	**CR**	Absence of any PSMA uptake on follow-up PET
		**PR**	≥ 30% decrease in summed SUVpeak without any new lesion
		**SD**	< 30% decrease or < 30% increase in summed SUVpeak without new lesion
		**PD**	≥ 30% increase in summed SUVpeak or new lesions
**RECIST**	[21]	**CR**	Disappearance of all target lesions
		**PR**	≥ 30% decrease in the sum of diameters of target lesions
		**SD**	Neither PR nor PD
		**PD**	≥ 20% or ≥ 5 mm increase in the sum of diameters of target lesions
		**Non-Measurable**	Lymph nodes < 1.5 cm of short axis, osteoblastic bone lesions or osteolytic lesions with soft tissue mass less than 10 mm, all other lesions < 10 mm
		**Non-Target PD**	Worsening of lesions that were not selected as "target lesions" at baseline — either because they were too small to measure precisely, or because they were qualitatively assessed (bone, pleura, peritoneal disease)

For the visual therapy assessment, bPSMA and iPSMA images were compared and assessed according to the following criteria: the Positron Emission Tomography Response Criteria in Solid Tumors (PERCIST) [18], the PSMA PET Progression (PPP)[8] with clinical data, the adapted Prostate Cancer Working Group Criteria (aPCWG4) without involving confirmatory follow-up scan, that was recommended in the original criteria [19], the occurrence of four new lesions as PD (aPPP4) without any confirmation with follow-up and visual RECIP 1.0 (RECIP Visual) [20]. Patients were categorized as either PD or non-PD.

For the radiological therapy response RECIST 1.1 criteria was used [21]. The CT component of the PSMA PET/CT was assessed using RECIST1.1 criteria [21]. Up to five target lesions (with a maximum of two per organ) are selected at baseline for quantitative assessment, provided they are measurable, defined as having a longest diameter of at least 10 mm, or 15 mm in the short axis for lymph nodes. Bone lesions without any soft tissue component and lymph nodules less than 15 mm in short axis were regarded as non-measurable lesions. Patients were categorized as either PD or non-PD.

PSA > 50% was defined as a reduction of at least 50% in serum prostate-specific antigen levels from the baseline (pre-treatment) measurement to the lowest observed value (nadir) during or after therapy within three months of follow-up.

### Endpoints

The primary endpoint of the study was to define the prognostic significance of different therapy response criteria for overall survival (OS). OS was calculated from the date of bPET until the latest follow-up or time of death.

### Statistical analyses

Statistical analyses and visualization of the data were performed using SPSS software version 25.0 (IBM Corp., Armonk, NY, USA) and R version 3.5.3 (The R Foundation, Vienna, Austria). Kolmogorov–Smirnov test was used to determine the compliance of variables with normal distribution, and statistical tests were chosen accordingly. Descriptive data were presented as the median (interquartile range (IQR)) for skewed parameters. Kaplan–Meier analysis was performed to estimate median OS with a 95% confidence interval (95% CI). To evaluate the concordance between interim PET–based response criteria and between imaging-based and biochemical response classifications, agreement analyses were performed using Fleiss’ kappa (κ). Differences in overall survival between patients with PD and those without PD, using different therapy assessment methods, were determined by the log-rank test. A Cox proportional hazards model was used to examine the association of individual parameters with overall survival, and hazard ratios (HRs) and 95% confidence intervals (CIs) were presented. The prognostic ability of each response criterion was assessed using Harrell's concordance index (C-index). Receiver operating characteristic (ROC) curve analysis was performed to evaluate the discriminatory ability of each response criterion for the prediction of mortality. Pairwise comparisons of area under curves (AUCs) were conducted using the DeLong test for correlated ROC curves. Two-sided p-values less than 0.05 were considered statistically significant.

## Results

### Patient characteristics

The median age was 72.5 (IQR 67–79). After developing mCRPC, patients had received median of three lines (IQR 2–4) of therapy before LuPSMA. The baseline median PSA of the cohort was 485.5 ng/ml (IQR, 221.8–822.8). Baseline patient characteristics are summarized in Table 2*.*

**Table 2:** Patient characteristics (N = 84)

	**Median (IQR)**		**N (%)**	
**Age at LuPSMA**	72.5	(67.0–79.0)		
≥ 60			80.0	(95.2)
< 60			4.0	(4.8)
**Time from diagnosis to LuPSMA (years)**	5.8	(3.5–10.1)		
**Alive at the end of follow-up**			14.0	(16.7)
**Local therapy to prostate**				
Radical prostatectomy			37.0	(44.0)
Radiotherapy			10.0	(11.9)
None			33.0	(39.3)
Not available			4.0	(4.8)
**Number of prior lines for mCRPC**	3.0	(2.0–4.0)		
**Systemic Therapy after ADT**				
Docetaxel			71.0	(84.5)
Cabazitaxel			29.0	(34.5)
Abiraterone			67.0	(79.8)
Enzalutamide			65.0	(77.4)
^223^Radium			5.0	(6.0)
Other			18.0	(21.4)
**ECOG performance status**				
0			35.0	(41.7)
1			36.0	(42.8)
2			9.0	(10.7)
Not available			4.0	(4.8)
**miTNM baseline tumor volume (mL)**				
Tumor volume of N1/2 disease	5.0	(1.3–20.1)	62.0	(73.8)
Tumor volume of M1a disease	9.7	(3.2–33.0)	58.0	(69.0)
Tumor volume of M1b disease	284.8	(70.9–864.1)	81.0	(96.4)
Tumor volume of M1c disease	7.9	(3.5–69.3)	35.0	(41.7)
Total tumor volume (PSMA-VOL)	356.8	(149.5–911.1)	84.0	(100)
**Gafita risk for overall survival**[22]				
Low risk			40.0	(47.6)
High risk			44.0	(52.4)

According to the nomogram developed by Gafita et al., 44/84 (52.4%) of patients were initially at high risk of short overall survival [22]. The Median OS for the entire cohort was 13.2 months (95% CI, 11.6–14.9 months). Patients likely to respond according to Gafita et al. nomogram lived statistically longer (14.8 [95% CI, 11.5–18.1] vs. 9.7 months [95% CI, 8.5–10.9], p = 0.03) [22].

### Therapy response using different criteria and inter-rater agreement

After a median of four cycles of LuPSMA (IQR 2–5), median of 24.3 GBq cumulative dose (IQR 14.9–32.9 GBq), 60 patients (71.4%) experienced any decline in PSA, and 42 patients (50%) experienced a decline of at least 50%.

According to RECIST 1.1, a total of 34/84 (40.5%) patients had non-measurable disease. According to RECIST 1.1, aPCWG4, PERCIST, PPP, RECIP qPSMA, RECIP SUV ≥ 4 and visual RECIP, 20 (40%), 41 (48.8%), 44 (52.4%), 47 (56%), 35 (41.7%), 37 (44%) and 31 (36.9%) of patients had PD, respectively.

Table 3 summarizes post-iPET treatments. Importantly, PD according to imaging-based criteria alone did not automatically lead to discontinuation of LuPSMA therapy. Among patients who demonstrated PD on iPET according to visual RECIP, those who received additional LuPSMA cycles had a shorter median OS compared with those who were switched to chemotherapy (2.3 months [95% CI, 4.4–13.4] vs. 5.5 months [95% CI, 1.3–22.8]). However, this difference was not statistically significant (p = 0.3).

**Table 3:** Comparison of all PET-based response criteria and subsequent treatments following iPET (n = 76 with available treatment data)

	**Response**	**LuPSMA (N(%))**	**Best Supportive Care (N (%))**	**Androgen receptor signaling inhibitors (N (%))**	**Chemotherapy (N (%))**
**PPP**	PD	23 (30.3)	10 (13.2)	0 (0.0)	8 (10.5)
	Non-PD	34 (44.7)	0 (0.0)	1 (1.3)	0 (0.0)
**aPCWG4**	PD	39 (51.3)	0 (0.0)	1 (1.3)	1 (1.3)
	Non-PD	18 (23.7)	10 (13.2)	0 (0.0)	7 (9.2)
**PERCIST**	PD	20 (26.3)	10 (13.2)	0 (0.0)	7 (9.2)
	Non-PD	37 (48.7)	0 (0.0)	1 (1.3)	1 (1.3)
**RECIP Visual**	PD	11 (14.5)	7 (9.2)	0 (0.0)	7 (9.2)
	Non-PD	46 (60.5)	3 (3.9)	1 (1.3)	1 (1.3)
**RECIP qPSMA**	PD	15 (19.7)	7 (9.2)	0 (0.0)	8 (10.5)
	Non-PD	42 (55.3)	3 (3.9)	1 (1.3)	0 (0.0)
**RECIP SUV4**	PD	15 (19.7)	8 (10.5)	0 (0.0)	8 (10.5)
	Non-PD	42 (55.3)	2 (2.6)	1 (1.3)	0 (0.0)

Substantial agreement was observed between RECIP qPSMA, RECIP SUV4 and visual RECIP (κ: 0.7, p < 0.001). There was fair to moderate agreement between PSA ≥ 50% and interim PET response criteria as follows: RECIP qPSMA (κ: 0.50, p < 0.001), RECIP SUV4 (κ: 0.45, p < 0.001), visual RECIP (κ: 0.36, p < 0.001), PERCIST (κ: 0.48, p < 0.001), PPP (κ: 0.36, p < 0.001) and for aPCWG4 (κ: 0.45, p < 0.001).

### Tumor response assessment and survival outcomes

PD patients had a significantly worse OS compared to non-PD patients according to RECIST 1.1 (HR = 5.0; 95%CI, 2.5–10.0; p < 0.001), PERCIST (HR = 2.9; 95%CI, 1.8–4.8; p < 0.001), aPCWG4 (HR = 3.6; 95%CI, 2.1–6.0; p < 0.001), PPP (HR = 3.9; 95%CI, 2.3–6.6; p < 0.001), RECIP qPSMA (HR = 2.7; 95%CI, 1.6–4.4; p < 0.001), RECIP SUV ≥ 4 (HR = 2.2; 95%CI, 1.6–3.6; p < 0.001) and visual RECIP (HR = 3.5; 95%CI, 2.1–6.0; p < 0.001) *(*Table 4*).*

**Table 4:** Comparison of all PET response criteria sorted by C-index. Criteria were assessed for accuracy of overall survival prediction under LuPSMA. Cox regression analyses provided hazard ratios and corresponding p-values

	**Response**	**N (%)**		**HR (95% CI)**	**p (Cox)**	**C-Index (95% CI)**
**PPP**	PD	47	(56.0)	3.9 (2.3–6.6)	< 0.001	0.68 (0.58–0.77)
	Non-PD	37	(44.0)	Ref		
**aPCWG4**	PD	41	(48.8)	3.6 (2.1–6.0)	< 0.001	0.67 (0.57–0.77)
	Non-PD	43	(51.2)	Ref		
**PERCIST**	PD	44	(52.4)	2.9 (1.8–4.8)	< 0.001	0.65 (0.54–0.75)
	Non-PD	40	(47.6)	Ref		
**RECIP Visual**	PD	31	(36.9)	3.5 (2.1–6)	< 0.001	0.63 (0.52–0.73)
	Non-PD	53	(63.1)	Ref		
**RECIP qPSMA**	PD	35	(41.7)	2.7 (1.6–4.4)	< 0.001	0.62 (0.51–0.74)
	Non-PD	49	(58.3)	Ref		
**RECIP SUV4**	PD	37	(44.0)	2.2 (1.6–3.6)	< 0.001	0.62 (0.50–0.73)
	Non-PD	47	(56.0)	Ref		

**Fig. 2:**
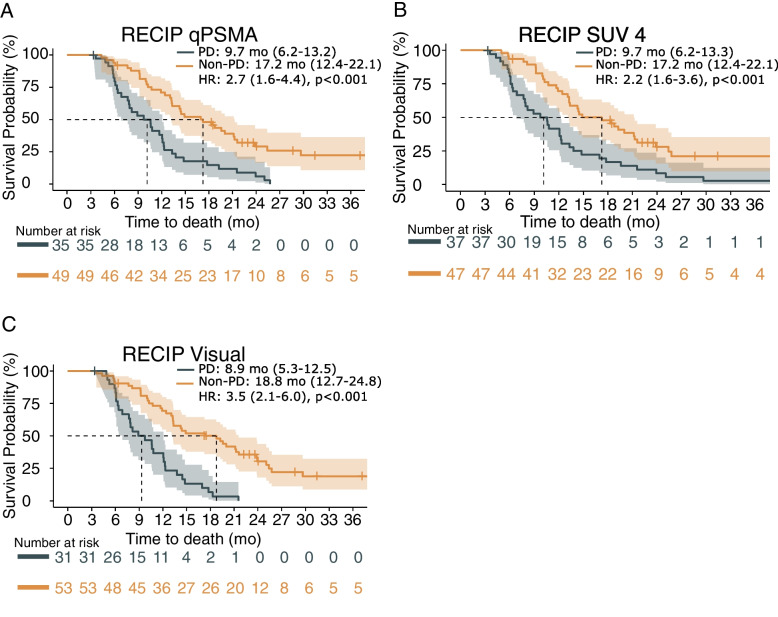
Overall Survival for different RECIP criteria with A) qPSMA threshold, B) with absolute SUV threshold (≥ 4), C) visual assessment. Median overall survival is shown for PD and Non-PD patients after 2 cycles of LuPSMA. Log-rank tests showed significant differences in survival between groups (all p < 0.001). Number of patients at risk in each time point is given below the plot. Cox regression analyses provided hazard ratios and corresponding p-values. Results are presented as median (95% CI) for OS and HR (95% CI) for Cox regression analyses

**Fig. 3:**
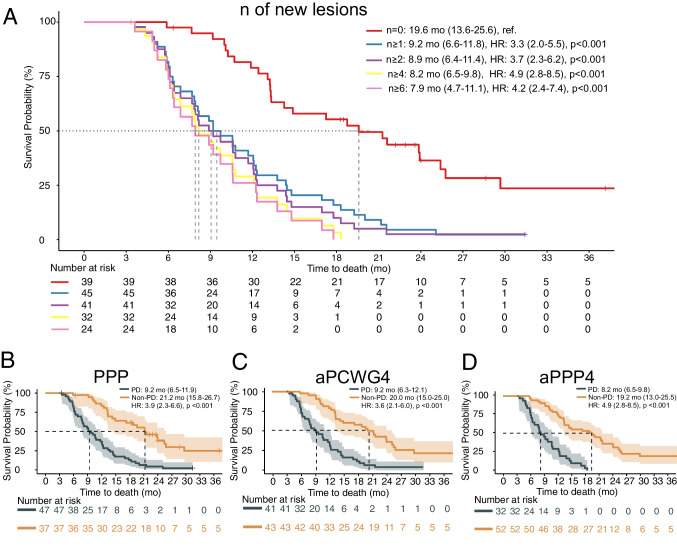
Overall Survival for different response criteria with A) number of new lesions, B) PPP, C) adapted PCWG4 requiring ≥ 2 new lesions, D) adapted PPP requiring ≥ 4 new lesions. Median OS for PD and non-PD patients after two cycles of LuPSMA is presented. Log-rank tests indicated significant survival differences between groups (all p < 0.001). Number at risk is shown below each plot. Cox regression analyses yielded hazard ratios with corresponding p-values. Results are presented as median (95% CI) for OS and HR (95% CI) for Cox regression analyses

**Fig. 4:**
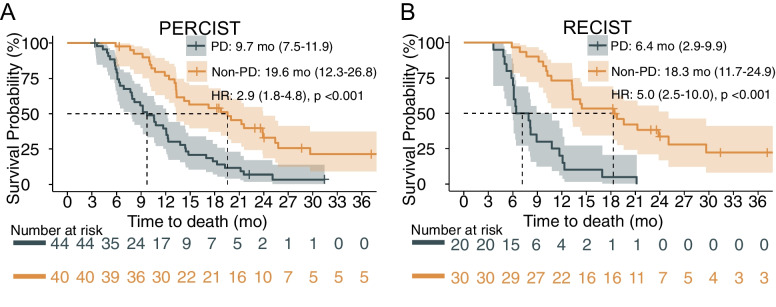
Overall Survival for A) PERCIST (n = 84), B) RECIST 1.1 (n = 50). Median OS for PD and non-PD patients after two cycles of LuPSMA is presented. Log-rank tests indicated significant survival differences between groups (all p < 0.001). Number at risk is shown below each plot. Cox regression analyses yielded hazard ratios with corresponding p-values. Results are presented as median (95% CI) for OS and HR (95% CI) for Cox regression analyses

Figure 2, 3 and 4 shows Kaplan–Meier curves of different therapy assessment criteria with hazard ratio analysed by Cox regression. Patients without any new lesion in follow-up had a longer survival, in comparison to patients with ≥ 1, ≥ 2, ≥ 4, and ≥ 6 lesions (median 19.6 months vs. 9.2, 8.9, 8.2, and 7.9 months, respectively). Figure 3A shows the Kaplan–Meier curves for new lesions.

Highest C-indices were observed for PPP: 0.68 (95%CI, 0.58–0.77), and aPCWG4: 0.67 (95%CI, 0.57–0.77) in comparison to PERCIST: 0.65 (95%CI, 0.54–0.75), visual RECIP: 0.63 (95%CI, 0.52–0.73), RECIP qPSMA: 0.62 (95%CI, 0.51–0.74) and, RECIP SUV ≥ 4: 0.62 (95%CI, 0.50–0.73*) (*Table 4*).* ROC curve analysis demonstrated comparable discriminatory performance among all evaluated response criteria, including PPP, visual RECIP, RECIP qPSMA, RECIP SUV ≥ 4, PERCIST, and aPCWG4. Pairwise comparisons of AUCs using the DeLong test revealed no statistically significant differences between any of the models (all p > 0.05).

**Fig. 5:**
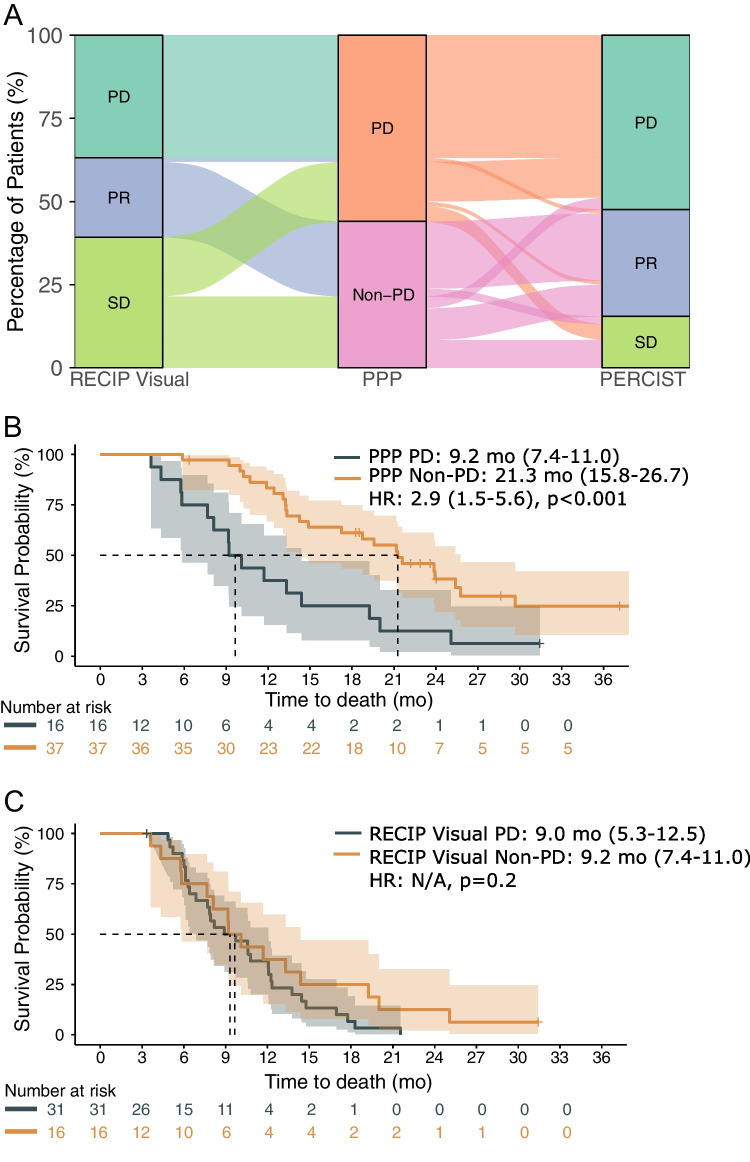
Comparison of PET-based response criteria: (A) Sankey diagram of RECIP visual, PERCIST, and PPP classifications; (B) OS curves for RECIP visual non-PD patients (n = 53); (C) OS curves for PPP PD patients (n = 47). Significant survival differences were observed between PPP PD and non-PD within RECIP visual PD (p < 0.001), but not between RECIP visual PD and non-PD within PPP PD (p = 0.2). Number at risk and Cox regression results are included. Results are presented as median (95% CI) for OS and HR (95% CI) for Cox regression analyses. Note: The PPP-based response assessment is binary, classifying patients based solely on the presence or absence of new PSMA-positive lesions. It does not define stable disease or partial response as response categories, distinguishing it from RECIP, RECIST and PERCIST criteria

Interestingly, among 53 patients with non-PD according to visual RECIP, 16 (30.2%) had PD according to the PPP. PD by PPP patients had a significantly shorter overall survival than those evaluated as non-PD with PPP (9.2 vs. 21.3 months, p < 0.001) (Fig. 5b)*.* Conversely, of the 47 patients with PD by PPP, 16 (34.0%) were classified as non-PD using visual RECIP evaluation. Notably, in the PPP PD group, there was no significant difference in outcome between PD and non-PD by visual RECIP (9.0 vs. 9.2 months, p = 0.2) (Fig. 5c)*.*

### Impact of PSMA radiotracer heterogeneity on therapy response assessment and overall survival

To evaluate the potential impact of radiotracer heterogeneity on therapy response assessment, we performed sensitivity analyses restricted to patients who underwent baseline and follow-up PSMA PET imaging with the same radiotracer (n = 49), as well as tracer-specific analyses ([⁶⁸Ga]Ga-PSMA-11, n = 26; [^18^F]PSMA-1007, n = 23). Overall, all six evaluated response criteria showed directionally consistent associations with overall survival in the tracer-consistent cohort (Supplementary Table 1). In the [⁶⁸Ga]Ga-PSMA-11 subgroup, robust and statistically significant associations with overall survival were observed for all response criteria (Supplementary Table 2). In contrast, in the [¹⁸F]PSMA-1007 subgroup, associations with overall survival were generally not statistically significant, with wide confidence intervals and unstable hazard ratio estimates for several criteria, likely due to high event rates and limited variability in survival outcomes (Supplementary Table 3).

## Discussion

In this retrospective study of patients with mCRPC treated with LuPSMA, we assessed treatment response using a variety of imaging-based criteria. Our findings highlight the prognostic value of these response measures and the variability among them, including between volumetric PET-based assessments and criteria focused on new lesion development. The discrepancies observed across criteria come with specific clinical implications for response classification and prognostication.

Consistent with prior studies, we observed a high rate of PSA decline following LuPSMA therapy, with more than two-thirds of patients experiencing any PSA reduction and nearly half demonstrating a ≥ 50% decline [23]. Moreover, post hoc exploratory analysis of VISION showed, that PSA decline has a prognostic value in terms of radiographic disease progression or death in mCRPC patients receiving LuPSMA [24]. While PSA kinetics remain a convenient biomarker for early therapy response, reliance on PSA alone may underestimate disease progression, particularly in the presence of discordant or non-measurable disease. In fact, retrospective analyses have shown meaningful discordance between biochemical and imaging responses. One study reported a 47% discordance between PSMA PET/CT and PSA responses in mCRPC, with PSMA PET/CT detecting progression in 31% of patients who had shown > 50% PSA declines [25]. Another study showed, the strength of agreement between PSA response and PET/CT response criteria was only fair (kappa 0.35) [26]. Similarly, in our cohort, agreement between PSA decline and PET-based responses was fair to moderate (Fleiss kappa between 0.36 and 0.50), highlighting the added value of molecular imaging in response assessment alongside PSA dynamics.

RECIP showed substantial agreement with other PET-based criteria and moderate agreement with PSA decline, but slightly lower prognostic performance (C-index: 0.62). Our results are similar to findings of Gafita and his colleagues with exception of higher C-index of RECIP criteria found by Gafita et al. (0.68 vs. 0.62 in our study) [20]. It was found that up to 86% of patients exhibiting mixed responses as well as 67% developing new lesions during LuPSMA therapy [27]. Gafita et al. suggested that relying solely on occurrence of new lesions to define PD might lead to premature treatment cessation [20]. In contrast, in our analysis, criteria explicitly incorporating new lesion detection, such as PPP and aPCWG4, were associated with higher hazard ratios and the strongest prognostic performance. Patients classified as PD by these criteria had significantly worse overall survival, and both PPP and aPCWG4 demonstrated the highest C-indices (0.68 and 0.67, respectively). Although formal statistical comparison of C-indices did not reveal significant differences between models, these findings suggest that the emergence of new lesions represents a particularly sensitive marker of aggressive disease biology.

There is clinical relevance of discordant classifications between criteria. In our cohort, 16 patients classified as non-PD by visual RECIP were found to have PD by PPP, and these patients had significantly worse survival compared to those classified as non-PD by both (9.2 vs. 21.3 months, p < 0.001). Conversely, within the PPP-defined PD group, patients classified as non-PD by visual RECIP had similar poor outcomes (9 vs. 9.2 months, p = 0.2), reinforcing the idea that new lesion detection provides relevant information, possibly more relevant than volumetric change. In survival analyses, we also found a clear, inverse relationship between the number of new lesions and OS. Patients with no new lesions had the longest survival (almost 20 months), while those with ≥ 2 or more new lesions had substantially worse outcomes (< 9 months). Michalski et al. also found that almost 70% of the patients had progression due to occurrence of ≥ 2 new lesions (27/32) or distinct increase in tumor volume (4/32) according to modified PPP criteria and had worse OS in comparison to Non-PD (7 vs. 29 months, p < 0.001) [28]. Murthy et al. also found that, among 11 of 20 patients who had new lesions on end-of-treatment PET, only one (9%) patient was classified as non-PD by RECIP 1.0 and had an OS of 9.7 months of OS [29]. While RECIP remains a valuable and standardized tool, particularly for centers with quantitative PET capabilities, our findings underscore the clinical relevance of new metastatic lesions. Their appearance serves as a straightforward and robust indicator of disease progression and poorer prognosis in patients receiving LuPSMA therapy.

While baseline and interim PET scans are often used to evaluate initial therapeutic response, they may not fully capture the complexity of disease dynamics, particularly in advanced stages where heterogeneous tumor biology and the emergence of mixed responses are common. The findings from Murthy et al. suggest that assessing end-of-treatment PSMA PET/CT scans, provides also reliable reflection of long-term survival outcomes, as it allows for a more comprehensive evaluation of tumor response, including the detection of new lesions and disease progression in comparison to baseline PET [29]. Moreover, as acquired resistance mechanisms become more prevalent in patients with advanced disease, understanding the patterns of progression, such as the emergence of mixed responses, is critical. Therefore, rigorous evaluation of PET-based response criteria after the fourth and sixth therapy cycles is warranted, as these later time points may offer a better understanding and more robust reflection of tumor dynamics and prognostic outcomes than early PET alone. In this intent, posttherapeutic whole body scans as well as SPECT/CT may play a valuable role in clinical decision-making. Several studies have demonstrated the prognostic value of changes in PSMA-VOL on posttherapeutic SPECT/CT imaging, which can be easily acquired unter LuPSMA [30–32]. Notably, the combination of posttherapeutic whole-body SPECT/CT and serum PSA levels has shown comparable accuracy to interim PSMA PET/CT after two cycles of LuPSMA therapy in early response assessment, providing preliminary evidence that SPECT/CT may serve as a cost-effective alternative to PET imaging without compromising clinical care quality [32].

An important consideration is how these findings may influence contemporary and future patient management. In our cohort, among patients who demonstrated PD on iPET according to visual RECIP, those who received additional LuPSMA cycles had a shorter median OS compared with those who were switched to chemotherapy, although this difference was not statistically significant. During the timeframe covered by this study, many patients received LuPSMA as a last-line therapeutic option, often with limited or no alternative systemic treatments available. Consequently, at our center, iPET-detected PD does not automatically lead to discontinuation of LuPSMA; decisions are made individually in a multidisciplinary setting, considering clinical status, PSA kinetics, and overall disease burden. In current practice, however, LuPSMA is increasingly being integrated into earlier lines of therapy, thereby expanding the clinical scenarios in which imaging-based PD may be encountered. This shift underscores the growing importance of understanding how to interpret PD detected on interim PET and how such findings should guide subsequent management decisions. Whether to change therapy immediately or continue LuPSMA despite PD in imaging remains an unresolved but clinically important question. Although the present study cannot definitively address these management dilemmas, it highlights the complexity of interpreting early imaging progression and offers a foundation for future research aimed at defining evidence-based strategies for handling imaging PD in the evolving treatment landscape. Prospective studies may help define which patients could benefit from continuing LuPSMA despite early imaging progression.

This study has several limitations. First, its retrospective design and relatively modest sample size may introduce selection bias and limit generalizability. Second, therapy response assessments and imaging segmentations were performed by a single reader, which, despite standardization, could introduce a bias. Third, two different PSMA radiotracers were used between baseline and interim PET imaging in a substantial proportion of patients. Differences in tracer biodistribution, uptake kinetics, and tumor-to-background contrast may influence lesion detectability, segmentation, and quantitative PET-derived metrics, particularly volumetric RECIP-based assessments relying on absolute SUV thresholds. This tracer heterogeneity therefore represents a major limitation and may have affected quantitative response classification. Our findings indicate that radiotracer heterogeneity may influence quantitative PSMA PET–derived response assessment and should be considered when interpreting therapy response metrics. Consequently, the application of volumetric PET criteria such as RECIP requires access to specialized software and technical expertise, which may not be available in all PET facilities. Future prospective, multi-reader, and multi-center studies are needed to validate these findings and establish standardized protocols.

## Conclusion

In conclusion, progressive disease during LuPSMA therapy, regardless of the PSMA PET/CT response criteria used, is consistently linked to shorter overall survival. Criteria based on new lesion detection (PPP, aPCWG4) show slight stronger association with overall survival than volumetric measures, highlighting new lesions as a clinically relevant marker of outcome. Standardized response criteria that incorporate reproducible longitudinal assessments are needed, and should be validated through large, prospective multicenter studies.

## Supplementary Information

Below is the link to the electronic supplementary material.

Supplementary file1 (DOCX 22 KB)

## Data Availability

The data that support the findings of this study are available from the corresponding author upon reasonable request.
