# Supplementary material for: PSMA-PET response under [177Lu]Lu-PSMA therapy: Comparison of RECIST, PERCIST, PPP, adapted PCWG4 and RECIP criteria
Source: Eur J Nucl Med Mol Imaging. 2026 Apr 24;53(9):5395–407. doi: 10.1007/s00259-026-07901-7 (PMC13314842; doi:10.1007/s00259-026-07901-7)
Supplement: Supplementary file 1 — Supplementary file1 (DOCX 22 KB) [file 259_2026_7901_MOESM1_ESM.docx]

**Supplementary Tables**

**Supplementary Table 1:** Comparison of all PET response criteria in the patients who underwent baseline and follow-up PSMA PET imaging with the same radiotracer (all cohort, n = 49; n=26 patients imaged with [⁶⁸Ga]Ga-PSMA-11 and n=23 patients imaged with [¹⁸F]PSMA-1007 pair). Criteria were assessed for accuracy of overall survival prediction under LuPSMA. Median overall survival is shown for PD and Non-PD patients after 2 cycles of LuPSMA. Log-rank tests showed significant differences in survival between groups. Cox regression analyses provided hazard ratios and corresponding p-values.

|  | **Response** | | **HR (95% CI)** | **p (Cox)** | **Median Survival (95% CI)** | **p (log rank)** |
| --- | --- | --- | --- | --- | --- | --- |
| **PPP** | PD |  | 3.8 (1.9-7.8) | <0.001 | 9.7 (7.4-12.0) | <0.001 |
|  | Non-PD |  | Ref. |  | 21.6 (15.2-28.0) |  |
| **aPCWG4** | PD |  | 3.5 (1.8-6.7) | <0.001 | 9.7 (5.6-13.8) | <0.001 |
|  | Non-PD |  | Ref. |  | 20.0 (14.4-25.6) |  |
| **PERCIST** | PD |  | 2.8 (1.5-5.3) | 0.003 | 9.7 (7.5-11.9) | 0.001 |
|  | Non-PD |  | Ref. |  | 18.8 (11.5-26.0) |  |
| **RECIP Visual** | PD |  | 3.4 (1.7-6.9) | <0.001 | 9.7 (6.3-13.1) | <0.001 |
|  | Non-PD |  | Ref. |  | 14.9 (7.7-22.1) |  |
| **RECIP qPSMA** | PD |  | 2.8 (1.1-7.0) | 0.03 | 7.9 (4.3-13.4) | 0.02 |
|  | Non-PD |  | Ref. |  | 16.9 (12.0-21.9) |  |
| **RECIP SUV4** | PD |  | 3.6 (1.9-6.9) | <0.001 | 8.9 (6.2-11.6) | <0.001 |
|  | Non-PD |  | Ref. |  | 17.2 (11.8-22.7) |  |

**Supplementary Table 2:** Comparison of all PET response criteria in the patients who underwent baseline and follow-up PSMA PET imaging with [⁶⁸Ga]Ga-PSMA-11 (n=26). Criteria were assessed for accuracy of overall survival prediction under LuPSMA. Median overall survival is shown for PD and Non-PD patients after 2 cycles of LuPSMA. Log-rank tests showed significant differences in survival between groups. Cox regression analyses provided hazard ratios and corresponding p-values.

|  | **Response** | | **HR (95% CI)** | **p (Cox)** | **Median Survival (95% CI)** | **p (log rank)** |
| --- | --- | --- | --- | --- | --- | --- |
| **PPP** | PD |  | 5.2 (1.9-14.2) | 0.001 | 8.1 (6.2-10.0) | 0.001 |
|  | Non-PD |  | Ref. |  | 25.4 (18.9-31.9) |  |
| **aPCWG4** | PD |  | 3.7 (1.5-9.2) | 0.004 | 7.6 (4.9-10.4) | 0.001 |
|  | Non-PD |  | Ref. |  | 20.0 (14.8-25.2) |  |
| **PERCIST** | PD |  | 3.2 (1.3-7.7) | 0.01 | 7.9 (4.9-10.9) | 0.01 |
|  | Non-PD |  | Ref. |  | 20.0 (14.5-25.5) |  |
| **RECIP Visual** | PD |  | 5.9 (2.0-17.2) | 0.001 | 7.9 (3.2-12.6) | <0.001 |
|  | Non-PD |  | Ref. |  | 20.0 (14.2-25.8) |  |
| **RECIP qPSMA** | PD |  | 2. 8 (1.4-4.6) | 0.004 | 7.9 (4.3-11.5) | 0.02 |
|  | Non-PD |  | Ref. |  | 17.2 (13.5-21.0) |  |
| **RECIP SUV4** | PD |  | 5.9 (2.0-17.2) | 0.001 | 7.6 (4.9-10.4) | 0.002 |
|  | Non-PD |  | Ref. |  | 20.0 (14.8-25.2) |  |

**Supplementary Table 3:** Comparison of all PET response criteria in the patients who underwent baseline and follow-up PSMA PET imaging with [¹⁸F]PSMA-1007 pair (n=23) .Criteria were assessed for accuracy of overall survival prediction under LuPSMA. Median overall survival is shown for PD and Non-PD patients after 2 cycles of LuPSMA. Log-rank tests showed significant differences in survival between groups. Cox regression analyses provided hazard ratios and corresponding p-values.

|  | **Response** | | **HR (95% CI)** | **p (Cox)** | **Median Survival (95% CI)** | **p (log rank)** |
| --- | --- | --- | --- | --- | --- | --- |
| **PPP** | PD |  | NA | 0.2 | 11.7 (8.8-14.6) | 0.2 |
|  | Non-PD |  | Ref. |  | 13.3 (13.2-13.4) |  |
| **aPCWG4** | PD |  | 3.7 (1.2-11.5) | 0.02 | 11.7 (9.8-13.6) | 0.02 |
|  | Non-PD |  | Ref. |  | 13.2 (3.9-22.7) |  |
| **PERCIST** | PD |  | NA | 0.3 | 11.7 (9.7-13.7) | 0.2 |
|  | Non-PD |  | Ref. |  | 13.3 (13.2-13.4) |  |
| **RECIP Visual** | PD |  | NA | 0.2 | 10.6 (8.3-12.9) | 0.1 |
|  | Non-PD |  | Ref. |  | 13.3 (11.6-14.9) |  |
| **RECIP qPSMA** | PD |  | NA | 0.2 | 10.6 (8.7-12.5) | 0.2 |
|  | Non-PD |  | Ref. |  | 14.4 (13.1-15.7) |  |
| **RECIP SUV4** | PD |  | NA | 0.1 | 10.6 (6.4-14.7) | 0.1 |
|  | Non-PD |  | Ref. |  | 14.4 (13.1-15.7) |  |

NA = Cox regression not estimable due to high event rates and nonsignifcance.
